# Genotoxicity study of Ethiopian medicinal plant extracts on HepG2 cells

**DOI:** 10.1186/s12906-017-2056-x

**Published:** 2018-02-01

**Authors:** Wubayehu Kahaliw, Bjorn Hellman, Ephrem Engidawork

**Affiliations:** 1 Department of Pharmacology, School of Pharmacy, College of Medicine and Health Sciences, University of Gondar, P. O. Box: 196, Gondar, Ethiopia; 2 Department of Pharmaceutical Biosciences, Division of Toxicology, Uppsala University, Box 594, 751 24 Uppsala, Sweden; 3 Department of Pharmacology and Clinical Pharmacy, School of Pharmacy, College of Health Sciences, Addis Ababa University, P. O. Box: 1176, Addis Ababa, Ethiopia

**Keywords:** Comet assay, Genotoxicity, Pterolobium stellatum, Otostegia integrifolia, Extracts

## Abstract

**Background:**

Most of herbal medicines are used without any standard safety and toxicological trials although common assumption is that these products are nontoxic. However, this assumption is incorrect and dangerous, so toxicological studies should be done for herbal drugs. Although *Pterolobium stellatum*, *Otostegia integrifolia* and *Vernonia amygdalina* root extracts are frequently used in Ethiopian traditional medicine, there are no evidences of their active toxic compounds. Therefore, we made an effort to assess probable genotoxic effect of these plant extracts on DNA of human hematoma (HepG_2_) cells using alkaline comet assay.

**Methods:**

Genotoxic effects of extracts were evaluated using single cell gel electrophoresis (SCGE) method on HepG_2_ cell. Regarding comet data, the average mean tail intensities (TI) from each individual experiment and treatment (usually at least 3 cultures/treatment) were pooled and the average mean TI was used as an indicator of DNA damage and the standard error of mean (SEM) as the measure of variance.

**Results:**

DNA damage in the form of comet tail has been observed for 1 and 0.5 mg/ml *P. stellatum* chloroform and 80% methanol extracts on HepG_2_ cells, respectively. The chloroform extract of *P. stellatum* showed increased tail DNA percentage in a concentration dependent manner. Comet tail length in the chloroform *P. stellatum* extract treated cells (1 mg/ml) was significantly higher by 89% (*p* < 0.05) compared to vehicle treated controls. The rest of test extracts seemed to be without genotoxic effect up to a concentration of 0.5 mg/ml.

**Conclusions:**

Our findings show that two extracts from one plant evaluated have a genotoxic potential in vitro which calls for a more thorough safety evaluation. Such evaluation should include other end-points of genotoxicity apart from DNA damage, and possibly also pure compounds.

## Background

Most of herbal medicines are used without any standard safety and toxicological trials although common assumption is that these products are nontoxic. However, this assumption is incorrect and dangerous, so toxicological studies should be done for herbal drugs. Although *Pterolobium stellatum*, *Otostegia integrifolia* and *Vernonia amygdalina* root extracts are frequently used, there are no evidences of their active toxic compounds. Therefore, we made an effort to assess probable genotoxic effect of these plant extracts on HepG_2_ cells using alkaline comet assay [1].

*Pterolobium stellatum* (Forsk.) Brenan. (Fabaceae) is also called *kenteffa* (Amharic). *Pterolobium stellatum* is a scrambling or climbing, rarely semi-erect, multi-stemmed shrub with recurved prickles, growing from 2 to 15 m tall. *Pterolobium stellatum* is widespread in Africa, where it is found from Sudan and Eritrea southwards throughout Central, East and southern Africa to South Africa, but not in Angola, Namibia and Botswana. It also occurs in Yemen [2]. Fresh leaves and roots are chewed for medicinal purposes for tuberculosis and related respiratory diseases, diarrhorea, epilepsy and neuralgia [3–5]. Boiled roots are also used to treat common colds, persistent cough (asthma), and spleenomegally [6].

*Otostegia integrifolia* Benth (Lamiaceae (Labiatae)), *Tinjute* (Amharic) is one of the plants used in Ethiopian traditional medicine [7]. The plant has insecticidal properties and is often used as fumigant for pots and houses [8]. Traditionally, it is used to treat diabetes mellitus, tonsillitis, uvulitis and hypertension, malaria, ascariasis and lung diseases [9–14]. There are more than 65 compounds that have been isolated from Otostegia. Particularly, compounds from *O. integrifolia*, *O. perisca* and *O. fruticosa* were pharmacologically important [15]. From the aerial parts of the plant, isolation of eight prefuranic and furanic labdane diterpenes together with iridoid glucoside was reported. These were otostegin A, otostegin B, 15-*epi*-otostegin B, preleoheterin, leoheterin, and related compounds, including leopersin C, 15-*epi*-leopersin C, ballonigrin, vulgarol, and 8-O-acetylharpagide. In addition, the essential oil and chloroform extract of air-dried leaves of *O*. *integrifolia* were investigated and a total of 40 constituents including monoterpenes, sesquiterpenes, diterpenes and their derivatives were identified [16].

*Vernonia amygdalina* Del. (Asteraceae) has a variety of names in different languages. It is referred to as *grawa* (Amharic); vernonia tree, bitter leaf (English). *Vernonia amygdalina* is a shrub or small tree of 2-5 m with petiolate leaf of about 6 mm in diameter and elliptic shape. The leaves are green with a characteristic odor and bitter taste. The plant grows throughout Africa including Ethiopia. It is drought-resistant and thrives in humid environments [17, 18]. It is used to treat tonsillitis, epidemic diseases, bacterial infections, cough, bleeding, gastrointestinal problems, tuberculosis, asthma, constipation, oxidative stress, helmintic infections, malarial infections, gastrointestinal disorders, loss of appetite, wounds, thrombi, diabetes mellitus, lipid disorders, and breast cancer [11, 19–31]. The methanol leaf extract was relatively safe with median lethal dose, LD_50_ ≥ 5000 mg/kg/ when single dose was administered orally to mice [32].

Phytochemical screening of *V. amygdalina* has revealed the presence of saponins, glycosides and tannins, which are known to be bioactive purgative principles. Flavonoids are also present in bitter leaf and have identified three flavones – luteolin, luteolin 7-O-beta-glucuronoside and luteolin 7-O-beta-glucoside. These flavones possess antioxidant activity and may play a beneficial role in cancer prevention and offer some protection against diabetes and atherosclerosis. The high content of the antioxidant vitamin C present in *V. amygdalina* leaves also account for the antioxidant activity [33].

Drug discovery programs adopted cell-based assay applications with increasing frequency because cell systems are often inherently predictive of in vivo responses. Potential chemical toxicity, metabolic degradation or impaired permeability can be addressed with simple cell-based systems. Engineered or phenotype-specific lines also can be exploited to screen for compounds that modulate specific signaling cascades or regulatory elements [34].

Two types of variations in the genetic material of somatic cells which might lead to aging are DNA damage and mutations. DNA damages consist of a variety of chemical disorders in polynucleotide structure of the double helix, such as pyrimidine, apurinic sites, cross-links, and both large and small chemical additions, named adducts. DNA damage has a basic role in most of human diseases including cancer. An accurate, fast and sensitive method is required to evaluate DNA damage in toxicity studies, this method should be able to monitor DNA repair properly [1].

The aim of this work was to evaluate the genotoxic effects of *P. stellatum* chloroform and 80% methanol extracts as well as *O. integrifolia* and *V. amygdalina* chloroform extracts on HepG_2_ cells. Cells were treated with a range of extract concentrations from 0.01 mg/ml to 1 mg/ml. Vehicle and catechol were used as negative and positive control respectively. The alkaline Comet assay was used to determine genotoxicity.

## Methods

### Plant collection

The roots of *O. integrifolia*, and *P. stellatum* were collected from an area near *Angereb* River, Gondar town, North West Ethiopia, about 730 km away from the capital, Addis Ababa. The root of *V. amygdalina* was collected from Bure town, North West Ethiopia, about 400 km far from Addis Ababa. The plants were authenticated by a taxonomist (Mr. Melaku Wondafrash) and a voucher specimen of each plant material was deposited at the National Herbarium, College of Natural and Computational Sciences, Addis Ababa University for future reference with voucher numbers Wk001, WK002 and WK004 for *O. integrifolia*, *P. stellatum* and *V. amygdalina*, respectively. The plants were cleaned from dirt and soil and dried under shade for 2 weeks. The plants were spread out and regularly turned over to avoid fermenting and rotting. The dried root parts of plants were grinded using 0.75 mm sieve size hammer type mill, while the dried leaves were pulverized using a wooden mortar and pestle. The powdered material was weighed using an analytical balance and stored at room temperature.

### Crude extract

The air-dried; powdered roots of *P. stellatum*, *O. integrifolia* and *V. amygdalina* were exhaustively extracted with chloroform using maceration technique. Maceration was carried out using one liter of the respective solvent for 72 h, with regular shaking. The mixture was filtered with whatman No. 42 filter paper (Whatman No.42, England) and the filtrate was kept at +4^o^c. The marc was macerated again in the same solvent two times and filtered. The filtrates were combined evaporated under reduced pressure on a rotary evaporator (Buchi Rota Vapor R-200) and dried in oven at 40^o^c (Gallenkamp, England).

In parallel, the air-dried and powdered roots of *P. stellatum* were soxhlet extracted with 80% methanol (4:1, methanol: water). The extracts were filtered and evaporated under reduced pressure on a rotary evaporator and lyophilized**.** The extracts were kept refrigerated and away from light.

### Selection of exposure concentrations

Before the main experiments, pilot studies of solubility and viability were done to find the appropriate exposure concentrations for the evaluation of DNA damage (data not shown). The solubility of the extracts was tested in different solvents using 0.5 mg/ml as the maximum concentration during exposure. *P. stellatum* chloroform and 80% methanol extracts were easily soluble in 99.5% ethanol where as *O. integrifolia* and *V. amygdalina* chloroform, extracts were soluble in DMSO at 50 mg/ml. The final concentration of vehicle (solvent) was non-toxic and did not exceed 1%. The viability of the cells after 3 h exposure was checked using the Trypan blue exclusion method. According to Tice et al. [35], cell viability below 70% is considered to indicate cytotoxicity and lower cell viability than that should be avoided when evaluating potential genotoxicity in the comet assay. Thus, 0.01 to 1 mg/ml concentrations were used.

### Cell culture and exposure

Evaluation of genotoxic effects of crude extracts were carried out using HepG_2_ cells. Cells were obtained from department of pharmaceutical biosciences, faculty of pharmacy, Uppsala University, Sweden. Cryo preserved cells were cultured in Dulbecco’s Modified Eagle’s Medium (DMEM) containing 10% fetal calf serum (FCS) and 1% pencillin-streptomycin. One T_75_ flask containing 1 × 10^6^ cells and two T_25_ flasks containing 3 × 10^5^ cells were incubated for 3 days under 5% CO_2_ at 37^o^c. After 3 days, the cell culture was washed with phosphate buffer saline (PBS) and warmed at 37^o^c. Then, 2 ml and 1 ml trypsin was added to T_25_ and T_75_ culture flasks, respectively, incubated under 5% CO_2_ for 5 min. Fresh medium was added to the flasks and pooled in to a falcon tube. Then, the combined culture was centrifuged at 1100 rpm for 5 min and the pellet re-suspended with 3 ml media. After determining cell count, 4 × 10^5^ cells were seeded to six well plates in triplicate and incubated under 5% CO_2_ at 37^o^c for 2 days.

After 2 days of incubation, the medium was thrown away, washed twice with PBS and the cells were treated with the extract. Extract treated and control cultures were incubated for 3 h under 5% CO_2_ at 37^o^c. After 3 h, the medium was thrown away, trypsin was added and neutralized. Neutralized culture was centrifuged in five different falcon tubes and 1 ml of medium was added to each falcon to use for next stages of the comet assay.

### Comet assay

Immediately after exposure and washing, the cells were put on ice until the slides for the comet assay were ready and the cell viability was determined using the trypan blue technique. Only cell suspensions with viabilities of more than 90% were used for determination of DNA damage.

Each experimental set up was based on three independent electrophoresis runs, and from each electrophoresis run (cell culture); three slides per treatment were prepared for the assay. The DNA damage was evaluated using the alkaline version of the comet assay following a slightly modified protocol of Tice et al. [35] and Singh et al. [36]. Immediately after cell viability determination, the cells were centrifuged twice with DMEM, 5% FCS and 1% pencillin-streptomycin. The cells were then re-suspended and mixed with low melting point agarose at 37^o^c and applied to microscope slides (Menzel-Gläser Diagnostika, Germany), which had been pre-coated with normal melting point agarose. Triplicate slides were applied for each extract and control. The slides were covered with cover slip and placed in cold plate for 15 min at 4^o^c. The slides were then uncovered and incubated in a lysis buffer (2.5 M NaCl, 100 mM ethylenediamine tetraacetic acid (EDTA), 10 mM Trizma base, 1% Triton X-100, 10% DMSO, pH 10 adjusted with NaOH) at 4^o^c for 1 h protected from light.

The slides were transferred to an electrophoresis chamber at 4^o^c where they were treated in the dark with an alkaline electrophoresis buffer (300 mM NaOH, 1 mM EDTA, pH > 13) for 40 min before subjected to electrophoresis (25 V and 300 mA) in darkness at 4^o^c for 10 min. After electrophoresis, the slides were washed for 15 min in a buffer (0.4% Tris, pH 7.5), dried and stored in closed containers until the day of image analysis.

GelRed was added (10 μl) to 50 ml distilled water in a cuvette and the slides were incubated in the cuvette for 30 min in dark. The slides were held in neutralization solution (pH 7.5) for 30 s in dark. The slides were then kept in a humidity chamber until detection. After staining of the slides, detection was performed with an Olympus BX60 fluorescence microscope with an AVT FireWire camera (Stingray; Allied Technologies, Germany), a CoolLED pE-100 excitation light (536 nm) and the software Comet Assay IV (Perspective instruments, UK). All slides were coded independently and scored blindly. Apparently dead cells (comets without distinct heads: “clouds”) and super imposed comets were not captured during the image analysis. Fifty cells per slide were examined. All tests and controls were used in triplicate and at least three experiments were performed, which gave 450 examined cells per treatment. The mean tail intensity (showing the percentage of DNA that had moved from the nucleus towards the anode during electrophoresis) was used as an indicator for the level of DNA damage. Relative fluorescence intensity of head and tail, normally expressed as a percentage of DNA in the tail was considered as the parameter for measuring DNA damage in this case. This parameter is linearly related to the break frequency and covers the widest range of damage [37].

### Statistical evaluation of data

The statistical analysis of all results was done using the Statistica 10 and GraphPad Prism 5 softwares and the level of statistical significance was set at *P* < 0.05. Regarding comet data, the average mean tail intensities from each individual experiment and treatment (usually at least 3 cultures/treatment) were pooled and the average mean tail intensity was used as an indicator of DNA damage and the SEM as the measure of variance.

Differences in average values between vehicle-treated control cells and extract-exposed HepG_2_ cells were evaluated using a two-tailed unpaired t-test for independent samples, assuming equal variance between the different average mean values.

## Results

**Fig. 1:**
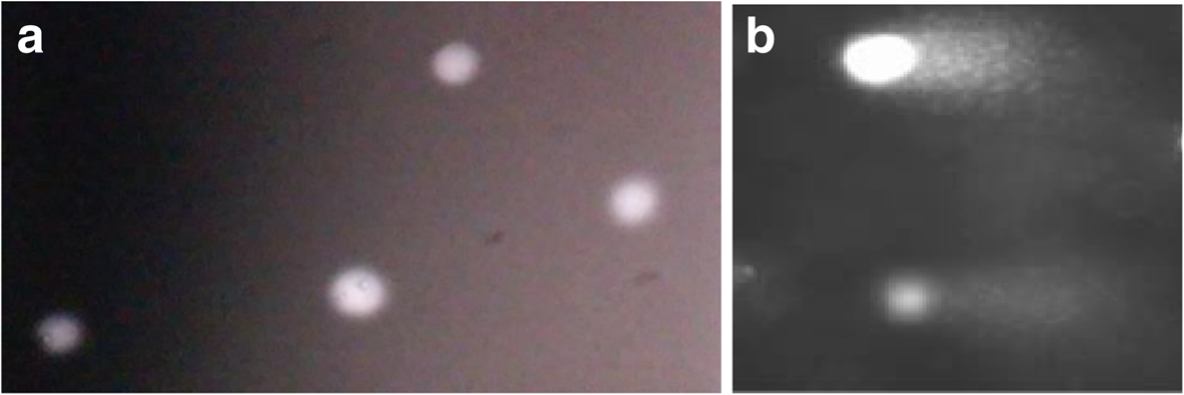
White spots [(**a**) comets without tails (**b**) comets with tails] of the comets that were detected in genotoxicity test. The software Comet Assay IV (Perspective instruments, UK) was used to analyse the images

White spots were detected in the genotoxicity assay of selected plant extracts as shown in Fig. 1. Comets with distinct heads and without tails (Fig. 1a) were vehicle treated non- migrated nuclear DNA from individual cells, whereas comets with distinct heads and tails (Fig. 1b) were extract treated and migrated ones.

Percent DNA in tail (tail intensity) in the DNA damage evaluation of the extracts along with the test concentrations are presented in Table 1. DNA damage in the form of comet tail was observed on HepG_2_ cells exposed to 1 and 0.5 mg/ml of *P. stellatum* chloroform and 80% methanol extracts (Table 1), respectively. The tail DNA percent ranged from 2.00 ± 0.09 to 4.03 ± 0.57 for *P. stellatum* chloroform extract treated sets at a concentration range of 0.01 to 1 mg/ml. The range was 2.61 ± 0.14 to 2.95 ± 0.02, 2.16 ± 0.26 to 3.02 ± 0.24, and 1.73 ± 0.10 to 2.90 ± 0.18 at test concentrations of 0.01 to 0.5 mg/ml for the chloroform extract of *O. integrifolia*, and for the chloroform extract of *V. amygdalina* (Table 1), respectively.

**Table 1:** Mean percentage DNA in tail (tail intensity) of different concentrations of extract treated and control HepG_2_ cells

No. of experiments	*P. stellatum* chloroform extract							*P. stellatum* 80% Methanol extract						*O. integrifolia* chloroform extract						*V. amygdalia* chloroform extract					
	Control		Concentration of extract (mg/ml)					Control		Concentration of extract (mg/ml)				Control		Concentration of extract (mg/ml)				Control		Concentration of extract (mg/ml)			
	Ethanol 1%	Catechol 3 mM	0.01	0.05	0.25	0.5	1	Ethanol 1%	Catechol 3 mM	0.01	0.05	0.25	0.5	DMSO 1%	Catechol 3 mM	0.01	0.05	0.25	0.5	DMSO 1%	Catechol 3 mM	0.01	0.05	0.25	0.5
1	2.59	–	2.04	2.04	2.38	2.56	–	2.21	5.03	2.89	2.72	2.90	2.97	2.64	5.60	2.62	2.51	3.25	2.31	2.64	3.76	1.64	1.82	2.07	2.99
2	1.80	5.88	2.13	1.95	2.93	2.00	–	2.05	5.18	2.51	3.07	2.21	2.90	3.08	5.18	2.16	2.43	2.54	3.00	2.63	3.36	1.61	1.57	2.59	2.55
3	1.30	3.20	1.82	2.53	2.31	2.72	2.89	2.37	5.08	2.42	2.38	2.30	2.97	2.57	3.88	1.71	2.97	3.27	3.00	2.60	4.54	1.93	1.22	1.78	3.15
4	1.47	3.16	–	1.54	1.72	2.67	4.51	–	–	–	–	–	–	–	–	–	–	–	–	–	–	–	–	–	–
5	3.47	5.82	–	–	3.55	3.94	4.70	–	–	–	–	–	–	–	–	–	–	–	–	–	–	–	–	–	–
Mean ± SEM	2.12 ± 0.40	4.52 ± 0.77	2.00 ± 0.09	2.02 ± 0.20	2.58 ± 0.31	2.78 ± 0.32	4.03 ± 0.57	2.21 ± 0.09	5.10 ± 0.04	2.61 ± 0.14	2.72 ± 0.20	2.47 ± 0.22	2.95 ± 0.02	2.76 ± 0.16	4.89 ± 0.52	2.16 ± 0.26	2.64 ± 0.17	3.02 ± 0.24	2.77 ± 0.23	2.62 ± 0.01	3.89 ± 0.35	1.73 ± 0.10	1.54 ± 0.17	2.15 ± 0.24	2.90 ± 0.18
*p*-value	1.00	0.022	0.819	0.827	0.399	0.239	0.030	1.00	0.0001	0.08	0.08	0.33	0.001	1.00	0.02	0.12	0.61	0.42	0.98	1.00	0.02	0.001	0.003	0.11	0.20

**Fig. 2:**
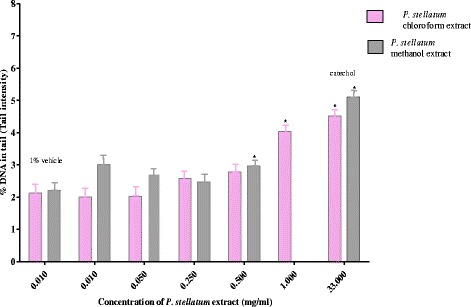
Genotoxic effect of *Ptrolobium stellatum* extracts on HepG_2_cells: cells were exposed for 3 h and DNA damage was monitored as an increase in percentage of DNA in the tail (tail intensity) after 10 min of electrophoresis in the comet assay. % vehicle = 1% ethanol (negative control) and 3 mM catechol (positive control). The means of percentage of DNA in tail for at list three experiments were compared with the vehicle control using T-test for independent samples

The chloroform extract of *P. stellatum* showed increased tail DNA percentage in a concentration dependent manner. It had an increased genotoxic effect after 3 h exposure at a concentration of 1 mg/ml compared to controls. Comet tail length in the extract treated cells (1 mg/ml) was significantly higher by 89% (*p* < 0.05) compared to vehicle treated controls. However, at lower concentrations no apparent genotoxic effect was observed. The extent of DNA damage at 1 mg/ml exposure was comparable to the damage induced by the positive control (3 mM catechol) (Fig. 2).

*Pterolobium stellatum* 80% methanol extract significantly increased DNA damage at a concentration of 0.5 mg/ml (*p* < 0.001) when compared to the vehicle treated cells, although the damage was not concentration dependent (Fig. 2). Concentrations lower than 0.5 mg/ml were not significantly associated with DNA damage.

**Fig. 3:**
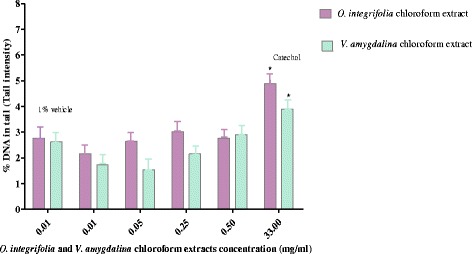
Genotoxic effect of *Otostegia integrifolia* and *V. amygdalina* chloroform extracts on HepG_2_ cells: cells were exposed for 3 h and DNA damage was monitored as an increase in percentage of DNA in the tail (tail intensity) after 10 min of electrophoresis in the comet assay. % vehicle = 1% DMSO (negative control) and 3 mM catechol (positive control). The means of percentage of DNA in tail for at list three experiments were compared with the vehicle control using T-test for independent samples

Treatment of cells with the choloroform extract of *O. integrifolia* did not show any detectable DNA damage at all exposure concentrations as compared to vehicle treated controls. However, the positive control demonstrated significant damage (*p* < 0.05) (Fig. 3).

Treatment of HepG_2_ cells with *V. amygdalina* at 0.01, 0.05 and 0.25 mg/ml concentrations showed tail DNA percentages 1.73 ± 0.10, 1.54 ± 0.17 and 2.15 ± 0.24 respectively. The values were lower than the tail DNA percentage of the negative control (2.62 ± 0.01), while the value (2.90 ± 0.18) at 0.5 mg/ml exposure was greater than the negative control. Treatment of HepG_2_ cells with the chloroform extract of *V. amygdalina* produced a significant decrease in percentage DNA in tail at 0.01 mg/ml (*p* < 0.001) and 0.05 mg/ml (*p* < 0.05) compared to negative controls. The decrease and increase observed at 0.25 mg/ml and 0.5 mg/ml, respectively, however, was not statistically significant (Fig. 3).

## Discussion

The persistent use of traditional medicine in African population and natural products growing use in developed countries has led to a resurgence of the scientific interest in their biological effects. The assessment of the efficacy and safety profiles of the medicinal plants should be based on scientific evidence-based approaches including the genotoxic profile of such plants. The short-term tests for genotoxicity are typically used to identify potential mutagens and carcinogens. According to the frequent use of *P. stellatum*, *O*. *integrifolia* and *V*. *amygdalina* and no evidences of their active toxic compounds, we made an effort to assess probable effect these plants’ extracts on DNA of hepG_2_ cells using the alkaline comet assay [1].

Direct interaction between a DNA-reactive agent and DNA is one of several pathways that may lead to primary DNA damage in which the major end-points such as DNA strand breaks (which could also reflect repair incisions) and alkali labile sites are measured in the comet assay [38]. The comet assay has expanded an internationally well-known method to assay DNA damage in various cell types during the last three decades. It is believed that the comet assay is still growing in use and has high potential to be used in clinical and cancer research [1].

Major constituents have been identified in extracts from *O. integrifolia* and *V. amygdalina*, but the potential genotoxicity of these constituents remains rather obscure. However, there is sparse data regarding the bioactivity, safety and major constituents of *P. stellatum*. It is therefore difficult to assume that genotoxic effect observed at 1 mg/ml chloroform and 0.5 mg/ml 80% methanol extracts of *P. stellatum* might be induced by a specific compound. At the antimycobacterial concentration (0.039 mg/ml), these extracts were without DNA damaging effect [39].

This in vitro study was done on HepG2 cell culture and there is a long way to extrapolate to in vivo and determine the amount of plant that would affect human hepatoma. It is important to perform an in vivo study to recognize the amount of the plant that should be consumed orally to create concentrations of the herb extract that is genotoxic for HepG2 cells. In addition in vivo study helps to conclude if the amount of herb which is normally consumed is equal to amount of herb that is needed to make genotoxic effect and on the basis of this case decide about that normal use are genotoxic or not. In our previous study [39], the oral LD_50_ was greater than 5000 mg/kg for *P. stellatum* chloroform extract. However, sub-chronic and chronic toxicity as well as in vivo genotoxicity should be evaluated to establish the range of concentrations for the safe use of this plant extract.

In previous studies the aqueous and hydro-alcoholic extracts of *E. amoenum* increased % DNA in tail significantly and concentration dependently indicating the genotoxicity of these plants and their capability of DNA damage which is comparable with our finding in *P. stellatum* chloroform extract. However, the chloroform root extract of *P. stellatum* in this study was more genotoxic than the aqueous and hydro-alcoholic extracts of *N. jatamansi* and aqueous extract of *E. amoenu* [1].

Methanolic extract of *P. stellatum* showed significant increase in percentage DNA in tail at 0.5 mg/ml and this concentration was less than that shown by chloroform extract. It is therefore fair to assume that the methanolic extract of this plant might contain higher amounts of genotoxic constituents than the chloroform extract. In addition, *O. integrifolia* and *V. amygdalina* chloroform extracts were without significant genotoxic effects at the test concentrations. Conversely, research has indicated that one of the chemical constituents of *O. integrifolia* called stigmasterol had anti-genotoxic effect. Therefore it may be useful in prevention of certain cancers, including ovarian, prostate, breast, and colon cancers [14].

Upon exposure of HepG_2_ cells to *V. amygdalina* chloroform extract, significant increase in percentage DNA in tail was not induced and hence the extract didn’t show genotoxic effect at all test concentrations. The percent DNA in tail decrease at and below 0.05 mg/ml may be attributed to the extract’s action to protect the cells from background damage at low concentrations. In the previous study [31], the leaf extract of *V. amygdalina* indicated dose-dependent increase in DNA damage, reaching statistically significant value at 2 mg/ml in MCF-7 cells. Therefore, the DNA damaging effect of the root extract of *V. amygdalina* in this study is in agreement with study done on its leaf extract.

The major constituents in the leaves of *V. amygdalina* are different types of saponins and various flavonoids such as luteolin, luteolin 7-O-beta-glucuronoside, luteolin 7-O-beta-glucoside and vitamin C. These flavones possess antioxidant activity and might play a beneficial role in protection against background DNA damage which was observed at 0.01 and 0.05 mg/ml exposure [33]. The dose dependent genotoxic effect (though not statistically significant) at test concentrations equal/higher than 0.25 mg/ml might be attributable to higher concentrations of saponins and/or pro-oxidant flavonoids in this extract [38].

## Conclusion

The present paper has shown that chloroform and 80% methanol extracts of *P. stellatum* induced significant DNA damage in HepG2 cells. This suggests that components in these extracts might interact directly with the DNA. Studies with crude extracts are appropriate because it is in this form they are used as traditional herbal medicines. Some of the constituents in such plants can be genotoxic, others can be anti-genotoxic. Our findings show that two extracts from one plant evaluated have a genotoxic potential in vitro which calls for a more thorough safety evaluation. Such evaluation should include other end-points of genotoxicity apart from DNA damage, and possibly also pure compounds.

## Abbreviations

ANOVA: Analysis of Variance
DMEM: Dulbecco’s Modified Eagle’s Medium
DMSO: Dimethylsulphoxide
DNA: Deoxyribonucleic Acid
EDTA: Ethylenediamine Tetraacetic Acid
FCS: Fetal Calf Serum
HepG_2_: Human Hepatoma Cells
PBS: Phosphate Buffer Saline
SCGE: Single Cell Gel Electrophoresis
SD: Standard Deviation
SEM: Standard Error Of Mean
TI: Tail Intensities
